# The physiotherapeutic “Variable Approach Technique”: an example of neuromotor adaptation conveyed by the neuromuscular spindle

**DOI:** 10.1002/ccr3.1339

**Published:** 2017-12-22

**Authors:** Maria Russo, Giuseppe Cultrera

**Affiliations:** ^1^ ATC^®^ Association Via A. Manzoni, 88/c 95014 Giarre Italy

**Keywords:** Manual therapy, neuromuscular spindle, postural control, proprioception, rehabilitation, variable approach technique

## Abstract

The subcortical systems control the proper functioning of the automatic substrate required for movement. Such a substrate is often underestimated to give attention to voluntary movement. By applying Variable Approach technique, it is possible to communicate with automatic systems through muscular spindles to achieve a more functional volunteer movement.

## Introduction

This case report charts the assessment and treatment of a patient who comes to the physiotherapist observation with ambulation issues allegedly due to spastic paraparesis. The goal of this study was to show the effects of an innovative technique based exclusively on the variable approach technique (VAT).

From a neurophysiologic point of view, VAT is based on two fundamental requirements: the functional organization of the central nervous system (CNS) and the muscle spindles. The CNS can be divided into two big systems, motor and sensory 1; each of them is constituted by several structures that are interconnected through hierarchic and parallel networks. In particular, through the subcortical systems activity, the parallel connections allow for the functioning of the nervous system even after traumatic or vascular episodes which lead to injury of the motor cortex (the latter being the main responsible for voluntary movements). All of this is strictly related to automatisms. Although their importance is often underestimated, those automatic movements represent the main way to motor learning and explicit movement. The complex cortical and subcortical systems networks allow for the integration of visual, vestibular, motor, sensory, and proprioceptive stimuli, in which integration is fundamental for the organization of spinal reflexes, pre‐existent motor schemes that can be adapted to the specific motor task through a mechanism called functional attitude 2. Thanks to this organization, the cortex can focus on superior cerebral functions and finalized mobility, leaving to the subcortical structures the task of handling automatic activities such as ambulation and posture. It is, indeed, the suitable base of postural activity that enables the actuation of a fluid, harmonic and functional finalized movement.

The goal of VAT is the establishment of a “dialogue” between implicit and functional components of the CNS. This dialogue takes place by means of specific requests addressed through a direct manual intervention on the patient musculature, where the muscle spindles are located, only receptors of the human body that are modulated from the CNS in his role of informer. Placed inside a capsule in the muscular womb, the muscle spindles fibers are sensitive to muscle length variations of the order of 0.01 mm. The afferent channel, connecting the muscle spindles to the spinal cord, is composed by specific nervous fibers, some of them devoted to the transfer of information about the speed of the length variation, others dedicated to give information on the entity of the variation; they are slowly adapting receptors responsible of a continuous discharge frequency until the muscular fibers are elongated/stretched. All the proprioceptive information travels along the posterior cord of the spinal cord to reach the cerebellum and other CNS integration structures to be elaborated together with other sensitive afferents. During muscle contraction, the spindles continue with their activity, due to the efferent fibers (γ fibers) which, starting from the spinal cord and going directly to the polar region of intrafusal fibers, cause the co‐contraction with the extrafusal fibers (co‐activation alpha‐gamma). Also during a rest phase, the spindle fibers are stretched enough for determining a constant contraction, named muscle tone. Those modulation mechanisms make possible for the muscle spindles to constantly “inform” the CNS about the position of each body segment in the space and the contraction degree of each muscle, with the aim of guaranteeing a harmonic and effective motor performance.

The variable approach technique is focused on the muscles, most extended receptive structure of the human body as well as the responsible of movements, which are “informed” with the goal of stimulating important and adequate responses (both automatic and voluntary) from the nervous system, and observe the ability of neuromotor adaptation of the patient.

It should be considered that on an everyday basis, many of our muscles are differently stimulated, but not just through stretching or shortening along the articular axes, or antigravity holds. In fact, our muscles are also prone to pressure and transversal traction solicitation, acting on the muscle itself in various directions, with different intensities and time spans.

In traditional physiotherapy, the common approach for the ambulation functional recovery and improvement always involves exercises in which lower limbs are engaged in. This happens for either neurocognitive rehabilitation, for example, that with Perfetti method 3, 4, or other more diffused rehabilitative methods, for example, Bobath 5, 6, 7. Even though in the latter technique, also other body parts are taken into account (like the torso) in the ambulation recovery process, the greatest amount of practices and manual actions involves always the lower limb in all his structural aspects and his role in “walking exercises,” both propaedeutic and explicit. As in the Bobath method, exercises are often suggested for preparing a stand‐up walking by arbitrarily subdividing a continuous action (like walking) in several separate tasks 4. As a result, these arbitrary tasks come to operate completely different from the way they would within the continuous action, and their separate training gives a poor contribution to the performance of the whole patient ability.

“The literature devoted to physiotherapy suggests that the partial training does not always lead to an improvement of the execution of the whole target task. For example, for exercising the ambulation, it is not that useful to repeatedly practice forward and backward step with one foot in a pendulum movement. Disconnected from the typical pace set up when “moving a step forward and pass to the propping phase, the repetition of the steps backward and forward modifies the entire ambulation dynamics” 8.

When it traditionally believed that the walking is dysfunctional especially in virtue of a balance alteration, once again stand‐up exercises are suggested, with more or less involvement of the lower limbs in terms of specific requests upon them. But often a walking deficit is caused by the disorganization of the torso and abdomen musculature 9, 10, 11, 12, 13 used in a stereotypic way which does not allow nor provide a variety of “adjustable” schemes indispensable for the lower limb balance, and so useful for the ambulation in general. As earlier mentioned, the variable approach technique works on the muscle with various stimuli and in different directions, but without necessarily including articular surfaces movements 14. This fact, joint to the practical execution procedure of the technique represents the main difference with other rehabilitative approaches cited so far. In traditional rehabilitation, the movement (intended as shift of a segment along the articular axe) is a constant. With the VAT, it is possible to address the efforts toward the reorganization of CNS functional systems, through complex but clinically logic stimulations, without articular movements and, in this specific case study, without standing‐up. Furthermore, the characteristics of the technique allow its precise description through, among others, several quantitative elements, giving to the reader the possibility of clearly understanding which action is undertaken, where, how, and for how long.

## Methods

### Subject

An Adult male who was 37 years old was born by breech delivery. Around the age of 13, there is a gradual accentuation of lower limbs stiffness, with occasional problems while propping the foot sole properly. At the very beginning, these issues were ascribed to the patient's laziness and reserved personality. The situation remained steady until the age of 22, when the patient decided to undergo medical examinations as a consequence of progressive worsening of his condition. First, he was given an electromyography and then he had a neurologic consult, in a hospital in Messina. Blood samples were taken for DNA screenings, but for three times, the test tubes went missing. In the same hospital, the patient was diagnosed with presumed “paraparesis.” He was given botulinum toxin for three times, at 6 months intervals; after this treatment, a worsening of his physical conditions was reported by the patient, regarding lower limbs stiffness, which negatively affected the walking. There is no official transcription of the above‐mentioned information, which was given verbally by the patient. Subsequently, he went to a physiotherapist who suggested to have a neurologic consult and walking analysis.

The first admission went from 18 November to 06 December 2008. Release diagnosis: “Paraparesis ndn. Bilateral neurogenic club‐foot.” Rehabilitative plan: control gait analysis at full dosage of Baclofen oral route. Result: improvement of speed and distance within the same time interval. Walking scheme substantially unchanged, shorter knee hyperextension time and greater hip extension were noticed during propping central phase. A successive hospital admission is envisaged, for follow‐up and specific multilevel treatment with BoNT‐A on the distal musculature of lower left and right limbs. A domiciliary therapy was suggested with 25 mg Baclofen.

The second admission went from 08 June to 25 June 2009. Release diagnosis: “Spastic Paraparesis ndn, ambulation problems.” Carried out: Baclofen test i.t. with 50 mg in bolus;

- N/M block (1 mL Botox) on the following muscle groups: GAM 30 U sx, dx; GAL 20 U sx, dx; RF 25 U sx, dx.
- EMS infiltrated muscles (electrostimulation).

Results: the treatment with Baclofen bolus i.t. gave a 2 points decrease on the MAS scale for all the muscle district, with spastic hypertone on lower limbs.

The multilevel BoNT‐A treatment resulted in neither hypertone reduction nor variation of the walking scheme. A domiciliary therapy was suggested with 25 mg Baclofen 1tab. × 3.

- At this stage, the patient was proposed with the implant of a Baclofen pump, which he refused.

A few months after, under a friend advice, he went to Rome to have a consult with a physiatrist. The physiatrist confirmed the paraparesis diagnosis and advised a rehabilitative plan with the “Perfetti” method. After 3 weeks of the aforementioned rehabilitative plan in Rome, with the same physiatrist, the patient came back in Sicily and continued the rehabilitation with Perfetti method for about 4 years.

Results reported from the patient: walking improvement in terms of speed and stability, as well as better overall balance. Given that no other improvements were produced using the Perfetti method, his therapist suggested to continue with the variable approach method. Before: he lives on his own, drives a car, 100% manual skills. Now: he lives on his own, drives a car, 100% manual skills.

Hobby: he goes to the gym, training just upper limbs and torso. He volunteers for an association dealing with blood donation (Avis).

### Recent medical history

In July 2016, the patient comes to our observation, after no more recovery and improvements were taking place during the previous year through the rehabilitation with “Perfetti” method. His therapist suggested to continue with the VAT method.

### Subject exercise program and evaluation

Evaluation of functional activities during the first session:

#### Movements and transfers

Stand up from sitting position: forward support needed;

sit‐down: generic manual support needed;

autonomy in postural passages from sitting to lay‐down position and viceversa; rolling present on both flanks.

The patient autonomously eats, dresses, and washes himself; sphincter control present; fair control while seated; fair control while standing.

#### Walking

The walking is possible with reception of homolateral peak load, followed by the upper torso projection forward and upward. This push is exploited to drag the homolateral hemilate forward. The patient chooses to use the cane on the right side, because it feels safer to him. There is no disengaging between torso and pelvis, and during forward advancement, the bust is kept in extension to move the pelvis forward and take the steps. Knee remaining extended during the oscillation phase. During the ambulation, it seems that the attention of the patient is focused on the upper torso, and the latter drags, the whole body. As a consequence, walking turns out to be slow, unstable, and carried out with significant exertion, and more than once, the patient could not keep his balance and fell to the ground. Very poor automatic walking components are present, with the movement being executed just through voluntary control.

#### Evaluation lower limbs while supine

Left and right limb: hip flexion at 60° angle, with significant fall in external rotation and abduction, with no ability of correcting it in adduction. No dorsal flexion of the foot, which appears rigid and clubfoot.

- Prone position: of present 30° of knee flection. During walking, some of the movements are not functionally used, which were present in supine position; autonomous postural passages.

#### Good language/communication and cognitive functions

The main goal of the exercise program was to improve the walking. The short‐term goals can be summarized as follows: recruitment of abdominal musculature and torso, with the aim of increasing the activation time, strength, and coordination of those districts. This should on the whole improve the walking quality.

#### Rehabilitative intervention

Treatment with “variable approach” technique, administrated for 1 h circa, three times per week, for an overall 15 session cycle.

### Description of the applied VAT

**Figure 1:**
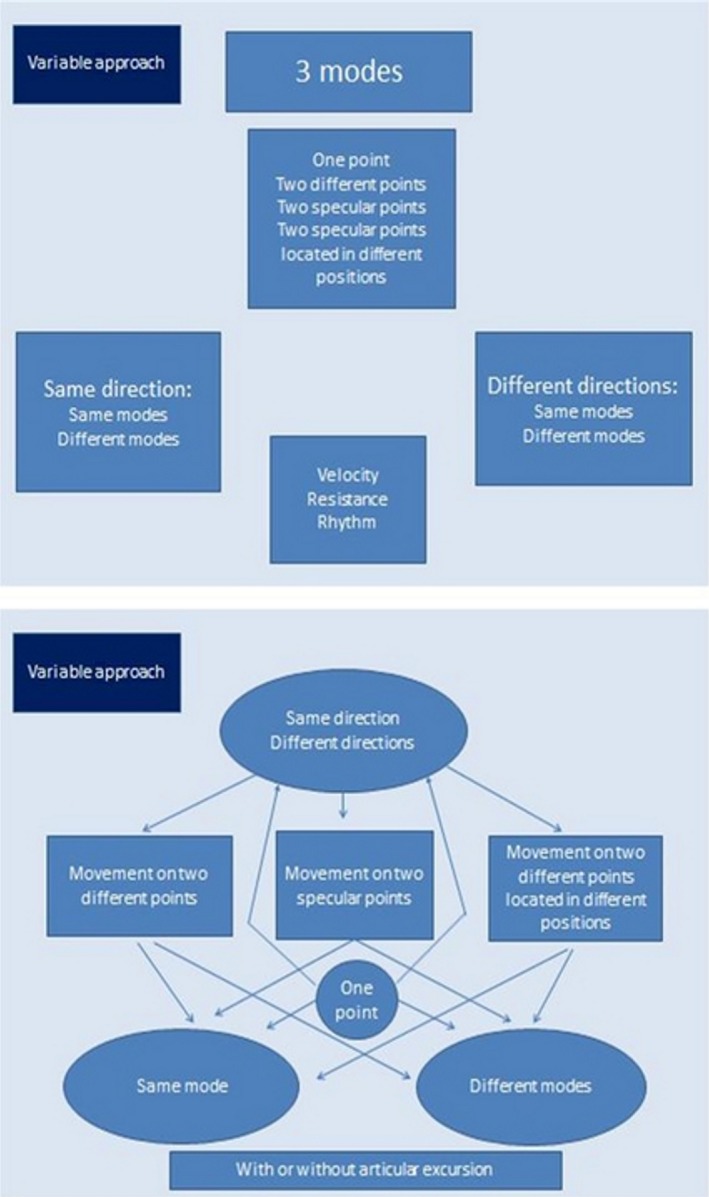
Schematic representation of the possible variants of application of the variable approach technique.

Synthetically, we can summarize, as shown in Fig. 1, the applicative aspects of the VAT.

It is possible to use the technique in one of the following ways:

1a: let the movement happen without any resistance;
1b: let the movement happen exerting resistance;
2: do not let movement take place;
3a: push and maintain the pressure;
3b: push and relax.

For any of the application scheme, some parameters can be changed if required, as:

1. velocity (low, medium, and high);
2. resistance (referring to resistance of the patient to the physiotherapist and vice versa);
3. rhythm.

Furthermore, the procedure can be applied:

1. on the same physical point;
2. on two different body points;
3. on two specular points;
4. on two specular points located in different position of the segments connecting them.

All of the above‐mentioned treatments can be performed with or without articular excursion.

It must be pointed out that it is possible to act on different directions in both a single point or various different ones.

Single‐point application:

- with or without articular excursion
- position: with or without support/propping
- procedure: let the movement happen, movement with resistance, do not let the movement happen, push forward, and maintain/relax
- directions with articular excursion: longitudinal, transversal, pressure, lifting, twisting, etc.…
- parameters: velocity, resistance, and rhythm.

Two points application:

Procedure analogous to the single‐point case, but with the following differences:

- the two points can be both muscular: same muscle, specular, or different muscles; otherwise they can be both articular specular of different, or even a mix of articular and muscular.

The two points can be supported, unsupported, or a combination of both.

In theory, there is a huge range of possible maneuvers and combinations of them. The choice of the specific combination of movements relies only on clinical considerations, not on the disease per se.

## Results

Here, we present the results about the enhancements that affect the patient treated through the following exercise sessions.

The Table 1 explains all the abbreviations used in this manuscript.

**Table 1:** Legend of abbreviations referring to the VAT

Abbreviation	Complete word
POS	Position
Z	Zone
MAN	Maneuver
FM	Free movement
MR	Movement with resistance
NM	No movement
PM	Push and maintain
PR	Push and release
DIR	Direction
RES	Resistance
V	Velocity
NP	Number of repetitions

### 1st session

Functional assessment. Collecting pictures of the patient ambulation. Administration of Berg Balance scale test (Table 2) 15.

**Table 2:** Berg's balance scale. 14‐item scale designed to measure balance of the older adult in clinical setting

Berg's balance scale items	1st session score	8th session score
1—Sitting to standing	3	3
2—Standing unsupported	4	4
3—Sitting unsupported	4	4
4—Standing to sitting	3	3
5—Transfers	3	4
6—Standing with eyes closed	4	4
7—Standing with feet together	4	4
8—Reaching forward with outstretched arm	3 (12.85 cm)	4 (25.7 cm)
9—Retrieving object from floor	0	4
10—Turning to look behind	2	4
11—Turning 360 degrees	0	1
12—Placing alternate foot on stool	0	0
13—Standing with one foot in front	0	0
14—Standing on one foot	0	0
Tot	30	39

Completion: time: 15–20 min; scoring: a five‐point scale, ranging from 0 to 4. 0 indicates the lowest level of function and 4 the highest level of function. Total score** = **56. Interpretation: 41–56 = low fall risk; 21–40 = medium fall risk; 0–20 = high fall risk. A change of 8 points is required to reveal a genuine change in function between two assessments.

The red color indicates items that modified their score.

The recorded measure in cm is the forward distance that the fingers reach while the subject is in the maximum forward tilt position.

Application of VAT:
POS: supine;
Z: abdomen: rectus, transverse, right upper and lower quadrant;
1 muscular point, 2 muscular points. Same procedure, same direction.
MAN: FM, NM;
DIR: medial, perp (perpendicular);
RES: patient;
Intensity: ++
V: L
RN: 10

### 2nd session

Assessment of lower limbs control and endurance in supine position, with flex hips and knees and with feet in support.

Standard position: 17 cm distance between the patient head and the upper bed edge, 12 cm feet distance.

Abduction and adduction control. Control of the dorsal flexion of the foot. Right leg showing bigger issues in controlling the adduction during abduction in eccentric. After a while, the control improves.

Home exercises: slow and simultaneous knees abduction and adduction.

Application of VAT:
POS: supine;
Z: lower abdominal quadrant, with flex hips and knees and standing feet;
1 muscular point.
MAN: FM, NM, MR;
DIR: posteroanterior perpendicular (pa‐perp);
RES: patience;
Intensity: +
V: L
RN: 10 for each procedure.
The session goes on with two muscular points.
Z: right upper and lower quadrant
Same procedure on the two points.
MAN: FM, NM, MR;
Same DIR: pa‐perp
RES: patient;
Intensity: +
V: L
RN: 10 for each procedure.
Subsequently, different procedures on the two points. Same DIR.
MAN:
FM: right upper quadrant; MR: left lower quadrant;
MR: right upper quadrant; FM: left lower quadrant;
FM: right upper quadrant; NM: left lower quadrant;
NM: right upper quadrant; MR: left lower quadrant.
DIR: pa‐perp
RES: patient
Intensity: +
V: L
RN: 10 for each procedure.

In addition: same procedure on two spots but different directions (pa‐perp, medial oblique caudal longitudinal, lateral oblique caudal longitudinal);

Z: right lower and upper quadrant
Two points: MAN = NM, FM, MR;

Same sequence with overball kept pushed between knees.

At the beginning, the patient is not able to perform the two tasks simultaneously; at half of the session time, he already gained more confidence and effectiveness in performing the tasks simultaneously.

### 3rd session

POS: supine.
Use of a band wrapped around the patient thighs, to help him maintain the position.
Two muscular points.
Z: right lower quadrant; DIR: MR in caudal longitudinal.
Z: left lower quadrant; DIR: FM in longitudinal caudal.
MAN: variation
Right: NM, DIR: caudal longitudinal; left: FM, DIR: caudal longitudinal;
Right: MR, DIR: pa; Left: FM, DIR: pa.
RES: patient;
Intensity: ++
V: L
RN: 10

Results: better response with respect to the second treatment (that was carried out without band).Short‐term goals: more selective and not massive responses, obtained through more precise information, focused on an improvement of his organization.

Remove the band and repeat the same treatment. Adaptations to the required maneuvers need more time to achieve the same sealing efficiency than with the band, but this endurance is at the end of the third overall treatment higher than the one obtained at the end of the second treatment.

### 4th session

Same procedure applied on the previous session, but without band assistance. Immediate response without losing legs endurance.

Walking re‐evaluation: the patient tells that now during ambulation, he can feel his knees flexing. This has not happened before the variable approach treatments, when they remained in extension while walking.

New treatment:
POS: supine;
Z: right upper and lower quadrant;
MAN: MR upper quadrant, FM lower quadrant;
DIR: pa‐perp;
RES: patient;
Intensity: +
V: L on upper quadrant, H on lower quadrant
RN: 10

Same sequence repeated on the left side. Best response on right side, compared to the left.

Z: right upper and left lower quadrants;
MAN: MR right upper quadrant; FM left lower quadrant;
DIR: pa‐perp
RES: patient
Intensity: +
V: L right upper quadrant, H left lower quadrant
RN: 10

Same sequence with inverse procedure on the two points.

Result: better control on the left side for all the sequences.

### 5th session

Re‐evaluation: repetition of the last sequence of 4th session. Result: retention of the response quality.

Application of VAT:
Z: right lower quadrant; left upper quadrant;
MAN: NM on the right and FM on the left;
DIR: right medial oblique caudal longitudinal, left pa‐perp;
RES: patient
Intensity: ++
V: M
RN: 10

### 6th session

Recording a video of the patient ambulation (Videos S1 and S2). Right and left knee flexion, in upright position, monopodalic and forward support. Better response for the right lower limb.Application of VAT on right and left hamstrings.

POS: prone
Single point:
Z: right hamstrings
MAN: NM
DIR: pa‐perp, caudal longitudinal, cranial longitudinal;
RES: patient;
Intensity: ++
V: M
RN: 10 for each direction.
Two specular points:
POS: supine;
Z: left and right hamstrings;
MAN: NM
DIR: various; equal and/or different on the two points
RES: patient;
Intensity: ++
V: M
RN: 10 for each direction.

Left knee flexion re‐evaluation; result: slight increase in the flexion movement.

### 7th session

Re‐evaluation of the response of neuromuscular organization.

POS: supine

Single point. Two points. Same direction, different procedures.

Z: abdominal wall; left and right upper quadrant; left and right lower quadrant;
MAN: FM, NM
DIR: anteroposterior perpendicular (ap‐perp)
RES
Intensity: ++
V: M
RN: 10 for each procedure.

Results:

- requests on a specific point: fair response ah 5th/6th repetition;
- requests in two points: with respect to the NM request, better response on both upper quadrants; with respect to FM request, better response on both lower quadrants.

The patient claims, as a result of the treatments undertaken so far, that his ambulation is safer (situation better experienced in case of rainy weather). The patient feels improved stability of lower limbs, which makes him less fearful of falling down and lessens the need for something to support himself with while he goes down the stairs.

### 8th session

Administration Berg Balance Scale test as re‐evaluation (Table 2).

POS: supine
Z: right upper quadrant and left lower quadrant
MAN: FM on the right, NM on the left
DIR: ap‐perp on the right, caudal medial longitudinal on the left
RES: patient;
Intensity: ++
V: M
RN: 10

Definite/clear response upon the fourth repetition.

POS: supine;
Z: right upper and left lower quadrants
MAN: NM on the right, FM on the left
DIR: ap‐perp on the right, caudal medial longitudinal on the left
Intensity: ++
RES: patient;
V: M
RN: 10

The same maneuver on the right.

POS: supine;
Z: left upper and right lower quadrants
MAN: NM on the left, FM on the right
DIR: ap‐perp on the left, caudal medial longitudinal on the right;
RES: patient;
Intensity: ++
V: M
RN: 10

The above ‐described sequence gives more effective responses/outcomes.

### 9th session

Functional assessment while seated: overlapping right leg on the left one and vice versa.

Application of VAT:
POS: supine, with flex right knee, hip external rotation and supported with a pillow, foot standing on the table plane
Single point:
Z: thigh adductor muscles
MAN: NM, MR, FM;
DIR: ap‐perp;
RES: patient;
Intensity: ++
V: L
RN: 10 for each procedure.
Z: Vastus medialis
MAN: NM, MR, FM
DIR: caudal medial longitudinal and caudal lateral
RES: patient;
Intensity: +++
V: L, M
RN: 10 for each procedure, direction, and speed.

### 10th session

Single muscular point:

Z: adductor muscles
MAN: FM, NM, PM;
DIR: caudal lateral longitudinal, caudal longitudinal;
RES: patient and physiotherapist;
Intensity: +++
V: M
RN: 10

### 11th session

The same treatment is repeated on the vastus medialis

Two muscular points:

Z: abdominal wall, right lower quadrant; right vastus medialis
MAN: NM right lower quadrant, NM vastus medialis
DIR: ap‐perp on right lower quadrant, caudal longitudinal on vastus medialis
RES: patient
Intensity: ++
V: M
RN: 10

### 12th session

The patient claims the actual “feeling” of his muscles for several hours after the previous session ended.

The previous treatment is repeated in the present session, with the following additions:

Two muscular points:

Z: left and right rectus femoris muscle
MAN: NM
DIR: caudal longitudinal
RES: patient
Intensity: +++
V: M
RN: 10

### 13th session

Single muscular point:

Z: left vastus medialis
MAN: FM, NM, MR
DIR: caudal lateral longitudinal, caudal longitudinal;
RES: patient
Intensity: ++
V: M
RN: 10 for each direction.

Two muscular points:

Z: right lower quadrant, left vastus medialis muscle
MAN: NM right lower quadrant, NM vastus medialis
DIR: ap‐perp on right lower quadrant, caudal lateral longitudinal and caudal longitudinal on vastus medialis
RES: patient;
Intensity: ++
V: M
RN: 10 for each procedure.

Result: increased hip flexion before leg crossing/overlapping.

### 14th session

The previous treatment is repeated in the present session, with the following additions

POS: seated

Two specular muscular points.

Z: left and right quadriceps
MAN: NM, MR, FM
DIR: caudal longitudinal, caudal medial longitudinal, caudal lateral longitudinal
RES: patient;
Intensity: +++
V: M
RN: 5 for each procedure and direction.

### 15th session

POS: seated

Two articular and muscular points

Z: upper torso, left and right lower quadrant (transverse abdominal muscle and internal/external oblique muscles)
MAN: NM on upper torso, FM on left and right lower quadrant
DIR: left and right rotation of upper torso; caudal longitudinal medial oblique and medial transverse of left and right lower quadrant;
RES: patient;
Intensity: ++
V: L
RN: 10 for each procedure and direction.

Result: Improved torso stability; in extension and during the whole hips flexion and limb crossing/overlapping, the torso remains steady (it does not fall backward).

## Discussion

**Figure 2:**
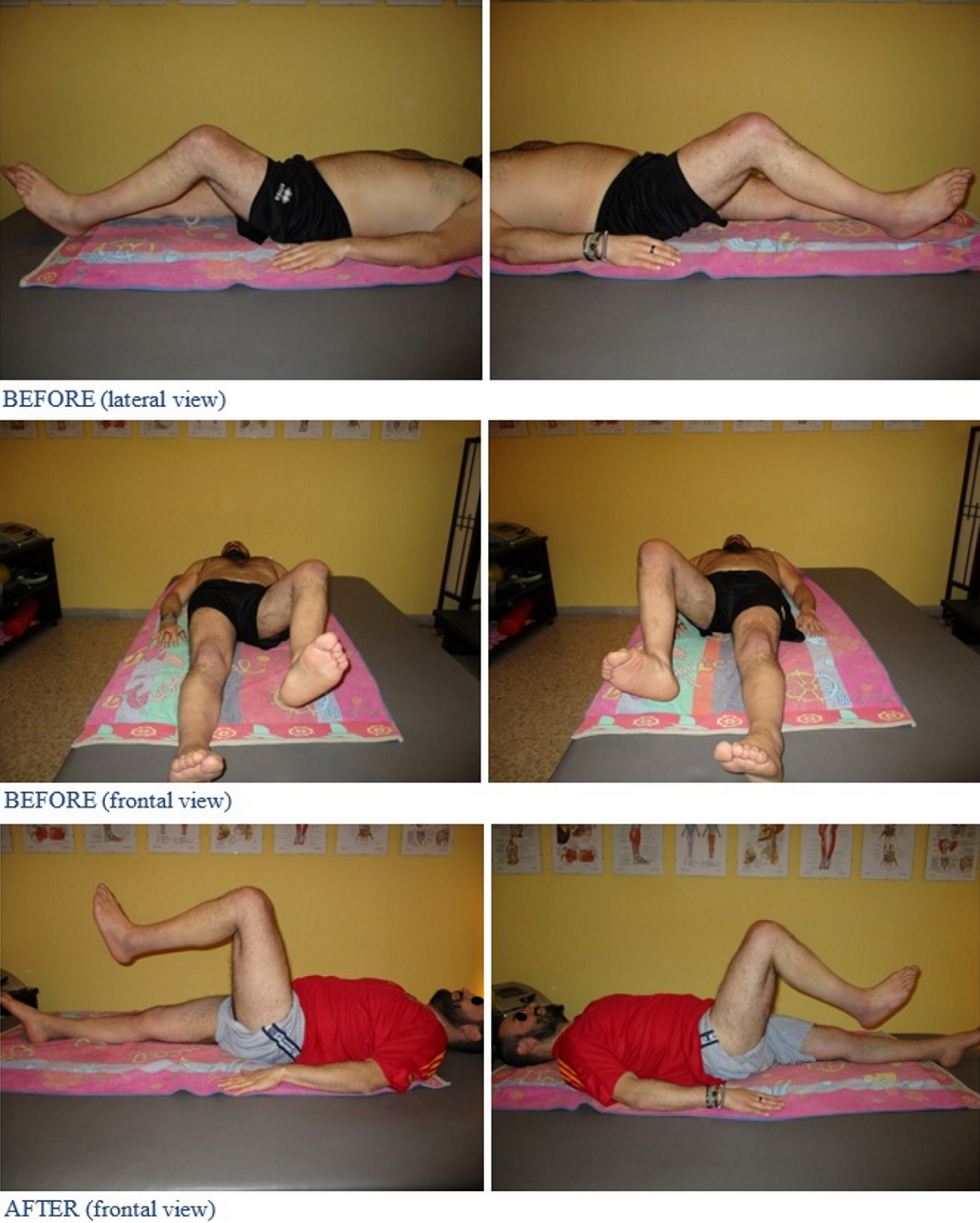
Enhancements enriched from the patient after 5th session. BEFORE: hips flexion possible only up to 45°, both right and left, absent abduction control. AFTER: possible abduction control with left hip flexion up to 90? and right hip flexion up to 70°.

This treatment cycle with the VAT produced several significant improvements. Starting from the 4th session, the patient claims a flexion of his knees upon walking. From the 5th session, an improvement took place on the hip flexion and endurance upon adduction as shown in Fig. 2.

Starting from the 7th session, the ambulation is more fluid and quicker. The patient can now prop almost the entire foot sole against the floor, and not just the tip (as he did before). The patient tells he is no more scared of stumbling, and he is now able to carry object with his hand while supporting on the cane with the other and walking (before he was not capable). Also, the ambulation in descent is safer.

The changes of direction are cleaner, quicker, and more effective, because he acquired greater gesture automatism. While changing direction, indeed, he needs less voluntary control and strength to contrast the movement inertia and so effectively modify the walking direction. Previously to the treatment with V.A. technique, if the patient had to turn left (or right), the adjustment of the direction would not take place in the timescale the patient predicted, because, due to inertia, he took other few steps forward before he was actually able to turn.

In the following paragraphs, other evidences are reported of walking improvements and automatic balance control.

While walking on uneven surfaces (e.g., gravel, dirt roads), the patient does not drag his feet, so he does not produce grooves or dust anymore.

He can take five steps from the garage door to the car with no need for a cane; before the treatment, he needed a continuous support, either using a cane or leaning on the floor.

**Figure 3:**
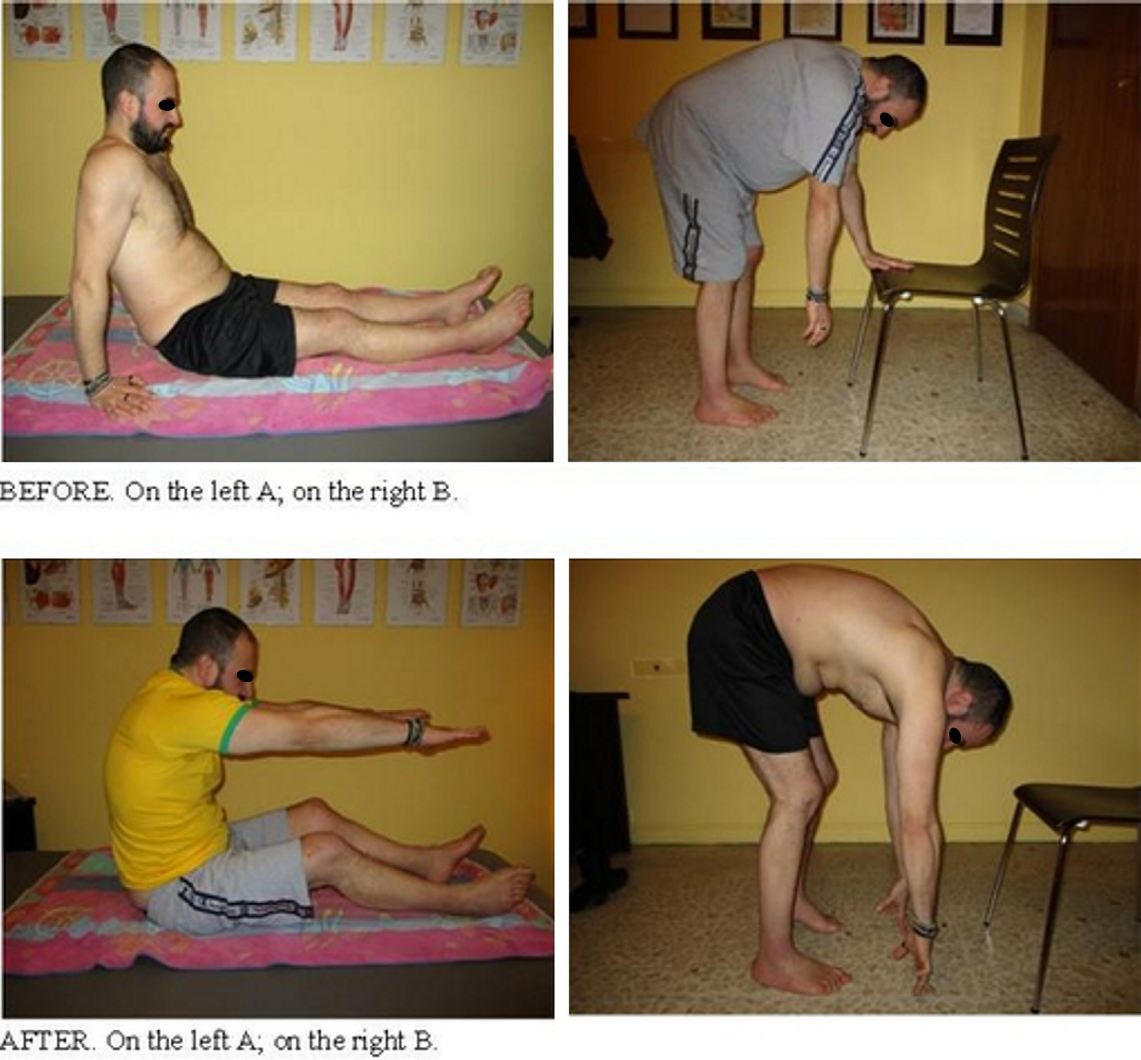
Additional enhancements enriched from the patient. BEFORE: (A) long‐sitting position possible only with a posterior support. (B) Flexion of the trunk possible only with an anterior support. AFTER: (A) long‐sitting position possible without any support. (B) Flexion of the trunk possible without any support.

He is able to pick up objects from the floor and go back to stand‐up position without need of sustains, while previously he had to first sit‐down or lean on a solid support (e.g., chairs, table) in order to lower himself and, more important, stand up (Fig. 3).

The forward flexion of the torso is improved, which allows the patient to touch the tip of his toes, and so wash them during a shower, by leaning his posterior to the wall. Before, in the same position, he could just reach his knees tibial spine. He then needed to get out of the shower and complete the feet cleaning while seated.

From the 7th session on, the patient claims he is capable of going upstairs while carrying shopping bags, needing much less time than it took before the VA therapy. Furthermore, he was often used to stumble while going upstairs, even if grounding himself with both hands on handrail and wall and without carrying any object. Now it stumbles rarely.

Previously, going downstairs just a half of the foot landed on the step, while the other half was outside it, so the patient was forced to steady himself by grabbing with both hands to the handrail and the wall. From the 7th treatment session, he can go downstairs by propping the entire foot sole within the steps area and leaning just on the handrail.

The Berg balance scale (BBS) was developed to measure balance among older people with impairment in balance function by assessing the performance of functional tasks. It is a valid instrument used for evaluation of the effectiveness of interventions and for quantitative descriptions of function in clinical practice and research. The BBS has been evaluated in several reliability studies. A recent study of the BBS, which was completed in Finland, indicates that a change of eight (8) BBS points is required to reveal a genuine change in function between two assessments among older people who are dependent in ADL and living in residential care facilities. At the 8th session, the Berg balance scale test was again administrated, with the following results: TOTAL SCORE: 1st session = 30; 8th session = 39.

Table 2. Berg balance scale. Description: 14‐item scale designed to measure balance of the older adult in a clinical setting.

Completion:

- Time: 15–20 min;
- Scoring: a five‐point scale, ranging from 0 to 4. “0” indicates the lowest level of function and “4” the highest level of function. Total Score = 56.

Interpretation: 41–56 = low fall risk; 21–40 = medium fall risk; 0–20 = high fall risk.

**Figure 4:**
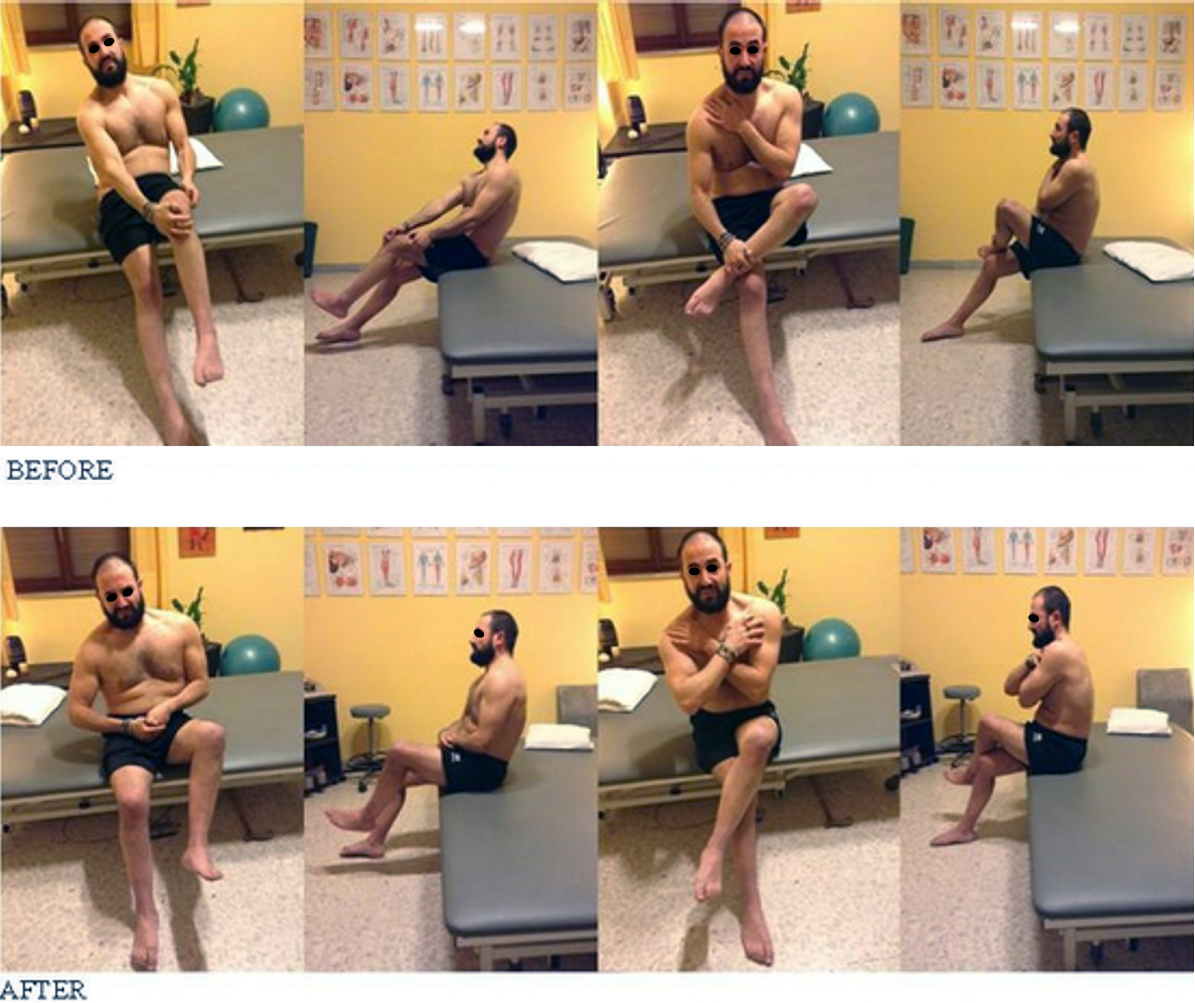
BEFORE: incomplete overlap of the left leg, with posterior fall of trunk. Possible only with the help of the upper limbs. AFTER: overlap of the left leg almost complete, with trunk in good position and without any help of the upper limbs.

**Figure 5:**
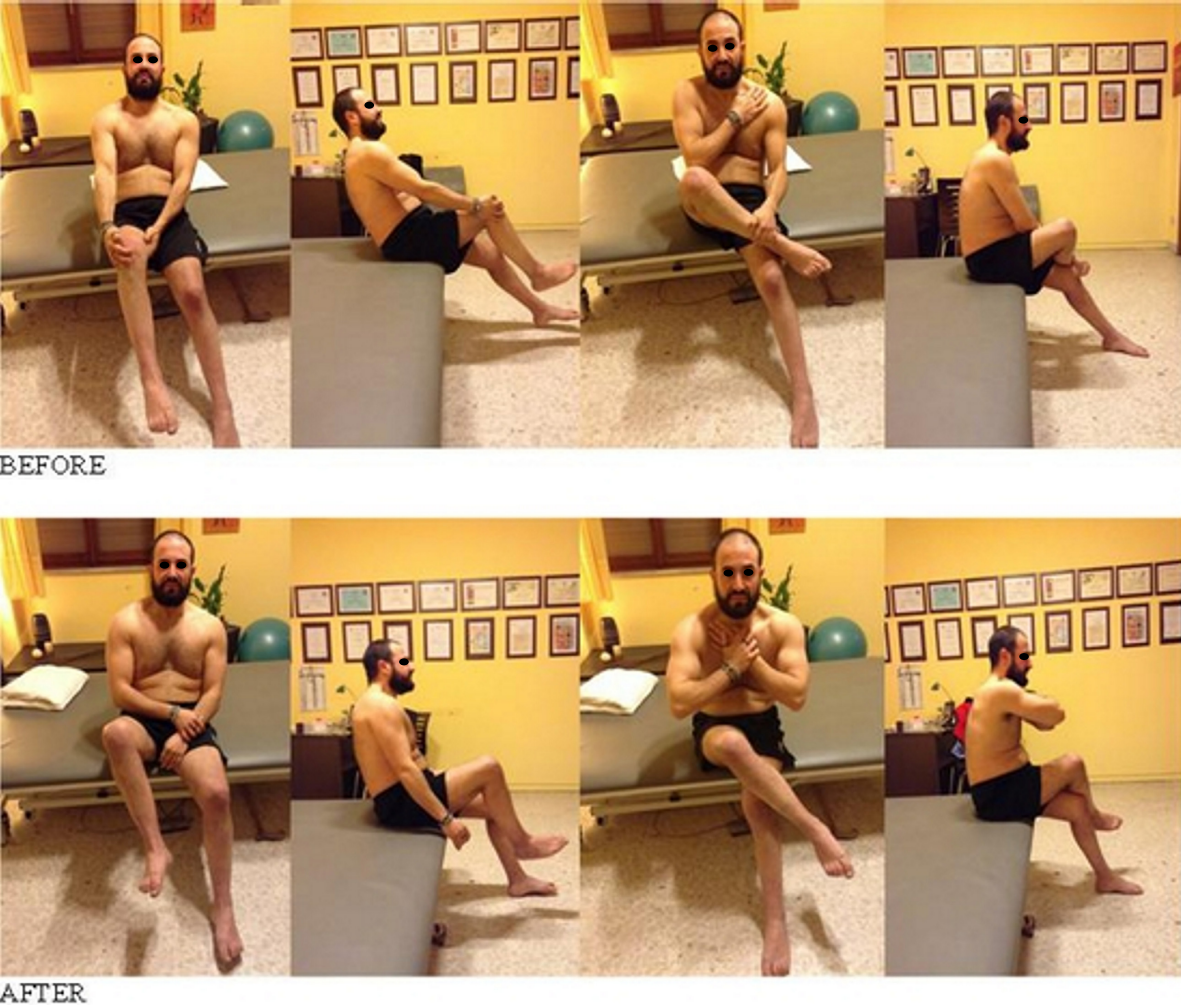
BEFORE: incomplete overlap of the right leg, with posterior fall of trunk and possible only with the help of the upper limbs. AFTER: overlap of the right leg almost complete, with trunk in good position and without any help of the upper limbs.

From the 13th session on, the patient is able to put on and off his trousers much easier and quicker (Figs. 4 and 5).

## Conclusions

Through the variable approach technique, the components of the automatic control of the movement emerged more evidently. Unlike other rehabilitative techniques, in this case, the increase in muscular strength has not been among the goals, nor were the muscular length and the articular excursion, but instead the efforts have been devoted to the functional organization of the patient movement, with special focus on the simultaneous activation of the latter's automatic and voluntary components.

When aimed to the movement reorganization, all the rehabilitative techniques take the afferent system as reference point. Opposite to traditional neurorehabilitation and even if it involves the afferent fibers, the variable approach technique gives plenty of possibilities of intervention, in virtue of its proper characteristic of allowing the precise clinic adjustment of the specific maneuvers during their execution, leading to a less approximate and more relevant response. Through characteristic and specific maneuvers, the reference system (i.e., afferent) can be administrated requests which can be adjusted as a function of the particular clinic conditions of the patient. In this way, the physiotherapist can clearly and extensively manage the information transferred to the patient, both in quantitative and (especially) qualitative.

In the case in object, the goal was the abdominal and torso musculature recruitment, looking for suitable activation timing and more significant and precise relations between the muscles. For the ambulation, no specific rehabilitative training has been undertaken, nor the direct activation of single components or phases of the walking process; we believe that it is a great neurophysiologic contradiction to subdivide a continuous action, as the ambulation is, in several discreet operations, the latter having naturally a different motor program compared to their subsequent and synergic execution during the whole action. When there is a great connection between two or more parts of an action, the latter has to be quickly corrected whether its execution is ineffective. Obviously, the due adjustment between each different component of the whole action cannot be exerted and learnt if the different parts are trained separately. As a consequence, mastering a single component of some actions does not guarantee the correct execution of the action as a whole. If just one component is trained (especially if on itself it shows different dynamics with respect to that when performing the global action), a different program has to be adopted, which is the one responsible of the solely execution of the specific part of action. The training of this program gives a contribution in the framework of learning that single action (differently structured), but does not give any in the context of the production of the whole movement. For these reasons, we did not work directly on the walking process, nor on its components, even though our final goal was, indeed, its improvement. We focused our attention on the possibility of recruiting more significantly the abdominal and torso muscles, in terms of timing and response quality. These muscles, prior to the variable approach treatment, presented an undeniable mass activity with poor selectivity, that while standing‐up turned the torso in a stiff block, unable to effectively neurophysiologically connect all the various torso components, and the torso itself to the lower limbs. Walking resulted as a consequence of a forced dragging and not as an automatic and integrated action of balancing and moving. The automatic aspect was evidently the poorest, and the ambulation was due only to a voluntary action, which is neurophysiologically costly, tiring, and unsafe. The correct and neat musculature recruitment determined a barycentre control that has led to a safer walking, more stable, and especially automatic, given that the patient himself claims that now he does not need to “think” about walking anymore. We are convinced that the quality and the type of information transferred to the patient could represent the key to success or failure of the plastic reorganization form the NS as a response to the received requests. The variable approach technique allows the physiotherapist a wide range of possibilities of providing, manage and manipulate the information, permitting an enrichment of the “automatic neuronal level” that translates in a more harmonic, organized, and efficient response of motor control. We would like to point out that also the pain, frequent within neurologic and orthopedic problematics, often relies and is sustained by a disorganization of the compensative response of the patient toward the trigger disorder. The variable approach technique has been proven to be really useful also in the case of acute pain in reducing this symptomatology, thanks to the possibility of not triggering the pain during the maneuvers administration, but obtaining instead an efficient neuromuscular reorganization and a consequent greater motor performance in terms of excursion, control, effectiveness, effort, and pain.

## Competing Interests

The authors declare that they have no competing interests.

## Authorship

MR: administered and verified the exercise sessions, followed the patients' enhancements, and collected data and wrote the manuscript. GC: developed the variable approach technique, supervised the exercise sessions and entire project, and wrote the manuscript.

## Supporting information


**Video S1.** Before Treatment

**Video S2.** After Treatment
